# CDK4/6 dual inhibitor abemaciclib demonstrates compelling preclinical activity against esophageal adenocarcinoma: a novel therapeutic option for a deadly disease

**DOI:** 10.18632/oncotarget.22244

**Published:** 2017-11-01

**Authors:** Juliann E. Kosovec, Ali H. Zaidi, Ashten N. Omstead, Daisuke Matsui, Mark J. Biedka, Erin J. Cox, Patrick T. Campbell, Robert W.W. Biederman, Ronan J. Kelly, Blair A. Jobe

**Affiliations:** ^1^ Esophageal and Lung Institute, Allegheny Health Network, Pittsburgh, PA, USA; ^2^ Department of Gastroenterological Surgery, Kanazawa University Hospital, Kanazawa, Ishikawa, Japan; ^3^ McGinnis Cardiovascular Institute, Allegheny Health Network, Pittsburgh, PA, USA; ^4^ Department of Oncology, Sidney Kimmel Comprehensive Cancer Center, Johns Hopkins Hospital, Baltimore, MD, USA

**Keywords:** esophageal cancer, Cdk4 protein, CDK6 protein, abemaciclib, preclinical drug evaluations

## Abstract

Esophageal adenocarcinoma (EAC) is a deadly disease with limited therapeutic options. In the present study, we determined the preclinical efficacy of CDK4/6 inhibitor abemaciclib for treatment of EAC. *In vitro*, apoptosis, proliferation, and pathway regulation were evaluated in OE19, OE33, and FLO1 EAC cell lines. *In vivo*, esophagojejunostomy was performed on rats to induce EAC. At 36 weeks post-surgery, MRI and endoscopic biopsy established baseline tumor volume and molecular correlates, respectively. Next, the study animals were randomized to 26mg/kg intraperitoneal abemaciclib treatment or vehicle control for 28 days. Pre and post treatment MRIs, histopathology, and qRT-PCR were utilized to determine response. Our results demonstrated treatment with abemaciclib lead to increased apoptosis, and decreased proliferation in OE19 (p=0.185), OE33 (p=0.048), and FLO1 (p=0.043) with anticipated downstream molecular inhibition. *In vivo*, 78.9% of treatment animals demonstrated >20% tumor volume decrease (placebo 0%). Mean tumor volume changed in the treatment arm by -65.5% (placebo +133.5%) (p<0.01), and prevalence changed by -37.5% (placebo +16.7%) (p<0.01). Pre vs post treatment qRT-PCR demonstrated significant inhibition of all downstream molecular correlates. Overall our findings suggest potent antitumor efficacy of abemaciclib against EAC with evident molecular pathway inhibition and reasonable safety, establishing the rationale for future clinical development.

## INTRODUCTION

The incidence of esophageal adenocarcinoma (EAC) has dramatically increased over the past four decades and currently represents the 6^th^ leading cause of cancer deaths worldwide [1]. The extremely lethal disease has an overall 5-year survival rate of only 18.8% due to the high rate of late-stage diagnoses and lack of effective therapeutic regimens [2]. Currently, standard-of-care treatment recommendations for locally advanced esophageal cancer include neoadjuvant chemoradiotherapy followed by surgery, demonstrating only minimal benefit with regard to prognosis [3, 4]. Therefore, significant advancements in screening strategies, risk stratification, and novel therapeutics are urgently needed to improve care and prognosis for patients.

**Figure 1:**
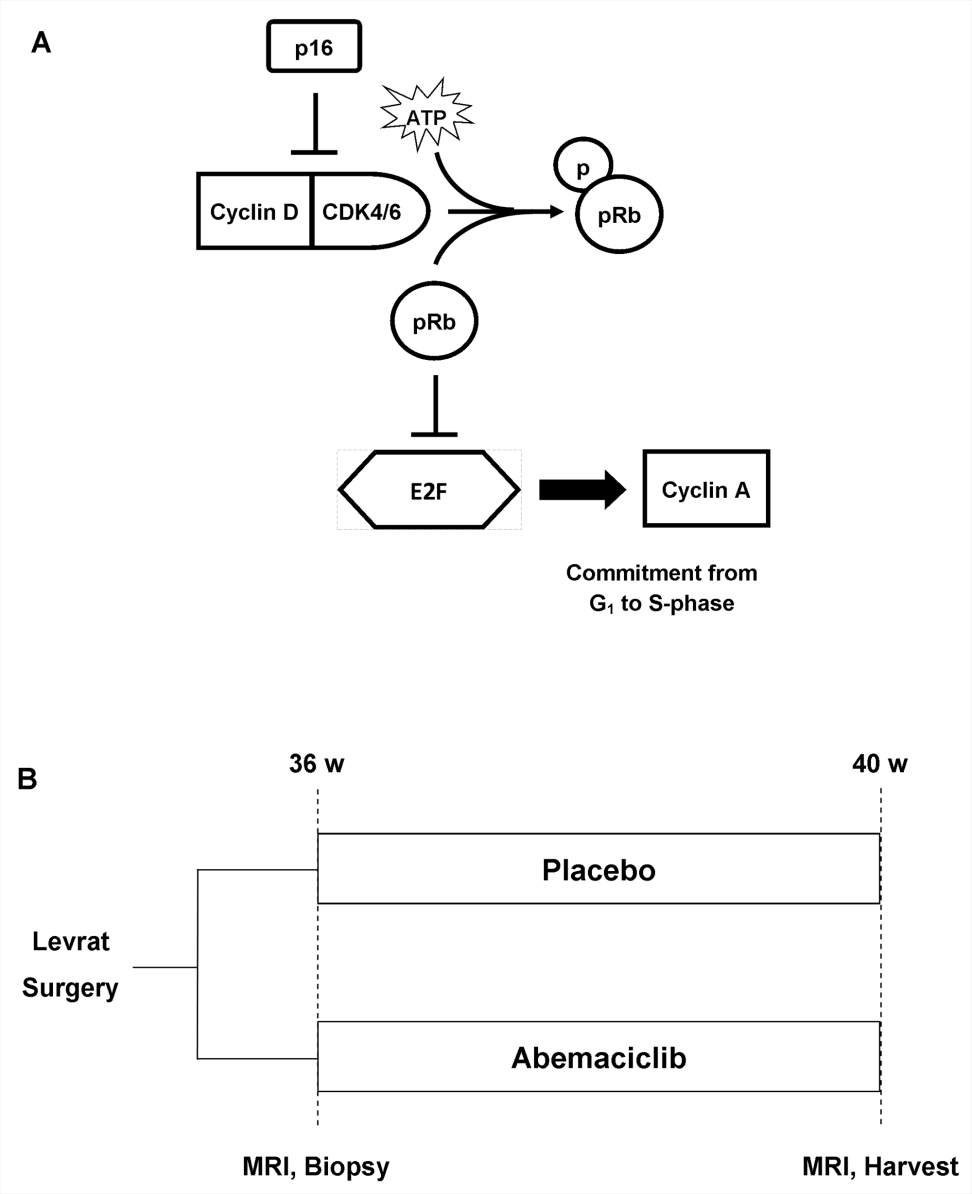
**(A)** CDK4/6 pathway. Tumor suppressor p16 normally inhibits Cyclin D-CDK4/6 complex, resulting in the release of E2F transcription factor and commitment to S-phase of the cell cycle. **(B)** *In vivo* study design. Modified Levrat surgery was performed on Sprague-Dawley rats to induce EAC. After 36 weeks, all animals received a baseline MRI scan and endoscopic biopsy and were randomized to control and treatment arms. At 40 weeks post-surgery, all rats received a final MRI and were euthanized. The entire esophagus was harvested 1cm distal to the anastomosis for histological and downstream molecular correlate analysis.

Recently reported analysis of The Cancer Genome Atlas (TCGA) indicates dysregulation of *CDKN2A*, the gene coding for the tumor suppressor *p16*, through deletion or epigenetic silencing in 81% of EAC/gastroesophageal junction cases and the associated significant upregulation of the cyclin dependent kinase (CDK) 4/6-cyclin D axis [5]. Specifically, amplification of the genes encoding CDK6 (7q21) and CDK4 (12q13) have been reported in 35% and 10% of gastroesophageal cancers, respectively [6]. Briefly, CDK4/6 binds to cyclin D and activates E2 transcription factor (E2F) via phosphorylation of the product of retinoblastoma tumor suppressor gene (p-pRb). Upregulation of the pathway leads to deregulation of the G1 checkpoint within the cell cycle, resulting in increased cellular growth and proliferation (Figure 1A) [7]. Additionally, CDK4/6 upregulation activates downstream expression of Forkhead box protein M1 (FOXM1), leading to evasion of cellular senescence [7, 8]. Previous studies have indicated that the pathway is progressively upregulated across the Barrett’s carcinogenesis spectrum and associated with poor prognosis in EAC [9, 10]. Although many first generation non-selective CDK4/6 inhibitors failed in clinical development due to toxicity, current CDK4/6 inhibitors in clinical trials are well-tolerated and have demonstrated potential efficacy in a wide variety of tumor types [11–13]. Recently published phase II and phase III clinical trial data demonstrated marked antitumor activity of abemaciclib in HR+/HER2- metastatic breast cancer and significantly improved progression-free survival and overall response rate versus standard-of-care therapy alone with a tolerable safety profile. [14, 15] These latest advances open the door for exploration into other solid tumors types, such as EAC.

The modified Levrat surgery of end-to-side esophagojejunostomy in rats is a validated preclinical model that causes chronic gastroduodenoesophageal reflux disease (GDER) to induce the development of *de novo* esophageal adenocarcinoma (EAC) through identical physiological and molecular processes that occur in humans [16, 17]. Numerous candidate therapeutic molecular pathways are conserved between human and rat EAC, including CDK4/6 [18]. The present study aims to evaluate the efficacy of the CDK4/6 dual inhibitor, abemaciclib, *in vitro* and *in vivo* through evaluation of tumor reduction and associated pathway regulation to set the stage for clinical trial development to treat locally advanced EAC.

## RESULTS

### In vitro

**Figure 2:**
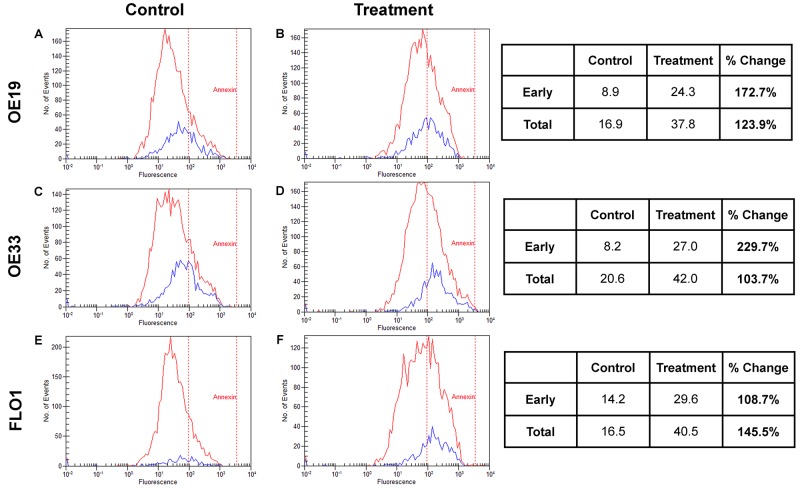
Apoptosis EAC cell lines OE19 (Panel **A** and **B**), OE33 (Panel **C** and **D**), and FLO1 (Panel **E** and **F**) were utilized to evaluate the effects of abemaciclib on apoptosis through flow cytometry analysis using Annexin-V and Calcein to stratify early and late apoptotic effects. With treatment, total apoptosis increased across all three cell lines by 123.9%, 103.7%, 145.5%, respectively. Early apoptosis also increased by 172.7%, 229.7%, 108.7%, respectively.

**Figure 3:**
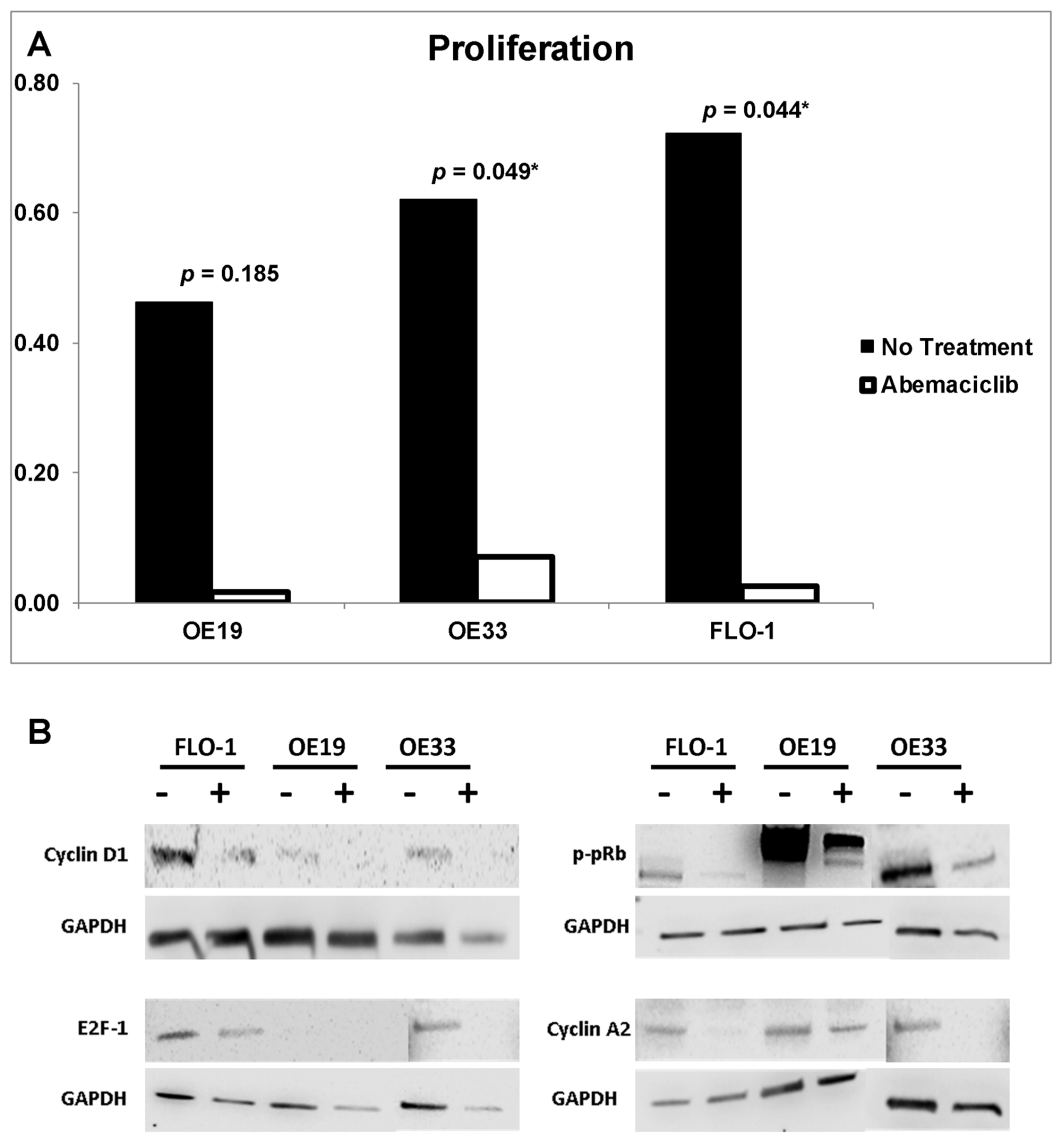
**(A)** Proliferation. Esophageal adenocarcinoma cell lines OE19, OE33, and FLO1 were utilized for proliferation analysis by BrdU ELISA using an ED50 of 10μM, 6μM, and 14μM, respectively. Proliferation significantly decreased in both OE33 and FLO1 after treatment with abemaciclib. **(B)** Western blot. Protein expression analysis revealed downregulation of Cyclin D1, E2F1, p-pRb, and Cyclin A2 across all cell lines due to treatment with abemaciclib (+) when compared to untreated (-).

ELISA-based WST-1 reagent toxicity test established the ED_50_ for OE19, OE33, and FLO1 as 10μM, 6μM, and 14μM, respectively. Flow cytometry showed Annexin-V-channeled total apoptosis increased with treatment by 123.9%, 103.7%, and 145.5% in OE19, OE33, and FLO1, respectively. Additionally, Calcein-channeled early apoptosis increased across all cell lines by 172.7%, 229.7%, and 108.7%, respectively (Figure 2). Proliferation analysis through ELISA-based BrdU assay demonstrated reduction with treatment of abemaciclib in OE19 (p=0.185), OE33 (p=0.048), and FLO1 (p=0.043), compared to non-treated cells (Figure 3A). Western blot analysis of treated cells demonstrated downregulation of cyclin D1, E2F1, p-pRb, and cyclin A2 across all cell lines (Figure 3B).

### In vivo

Mortality rate post-randomization in the treatment cohort was 18.75% (n=6), compared to no mortality in the placebo cohort. Causes of mortality included severe peritonitis (n=2), general morbidity (n=2), tumor obstruction (n=1), and inconclusive (n=1). Additionally, major health complications during the treatment window included peritonitis (placebo 12%; abemaciclib 94%), diarrhea (placebo 4%, abemaciclib 81%), and general morbidity (placebo 4%, abemaciclib 41%) (Table 1). Overall, 51 animals completed the study, including 26 abemaciclib and 25 placebo animals, respectively. One animal from the placebo arm and two animals from the treatment arm were excluded from overall MRI analysis due to poor quality 36-week or 40-week scans. Additionally, five animals from placebo and two animals from treatment were excluded from volumetric analysis due to negative EAC status at the time of randomization. Lastly, three animals in the treatment group and four animals from the placebo group were excluded from pre versus post treatment RT-qPCR analysis due insufficient pre-treatment biopsy tissue. The respective animals were still included in endpoint post-treatment gene expression comparison of placebo and abemaciclib cohorts.

**Table 1:** (A) Abemaciclib complications and mortality. Of the 32 animals receiving treatment, 41% displayed general morbidity, 81% had at least one episode of diarrhea, and 94% revealed peritonitis upon necropsy. Of the animals with peritonitis, 28% were severe. 6 animals were either found dead (n=2) or euthanized (n=4) prior to the endpoint of the study due to severe health complications. (B) Placebo complications and mortality. Out of 25 animals, 4% showed signs of general morbidity, and 12% demonstrated mild peritonitis. All animals survived until the endpoint of the study

ABEMACICLIB			PLACEBO		
Complication	# of Animals (n = 32)	Additional Comments	Complication	# of Animals (n = 25)	Additional Comments
**General Morbidity**	**13 (41%)**		**General Morbidity**	**1 (4%)**	
**Diarrhea**	**26 (81%)**	Mean = 3.89 days; Range = 1-10 days	**Diarrhea**	**1 (4%)**	
**Peritonitis**	**30 (94%)**	Severe = 8 (28%); Mild = 22 (72%)	**Peritonitis**	**3 (12%)**	Mild = 3 (100%)
**Penile Prolapse**	**2 (6%)**		**Penile Prolapse**	**0 (0%)**	
**Respiratory Infection**	**2 (6%)**		**Respiratory Infection**	**0 (0%)**	
**Found Dead**	**2 (6%)**	Severe Peritonitis = 1 (50%)	**Found Dead**	**0 (0%)**	
**Euthanized Prior to Endpoint**	**4 (13%)**	Severe Peritonitis = 1 (25%) Large Tumor = 1 (25%) General Morbidity = 2 (50%)	**Euthanized Prior to Endpoint**	**0 (0%)**	
**Endpoint**	**26 (81%)**		**Endpoint**	**25 (100%)**	

**Figure 4:**
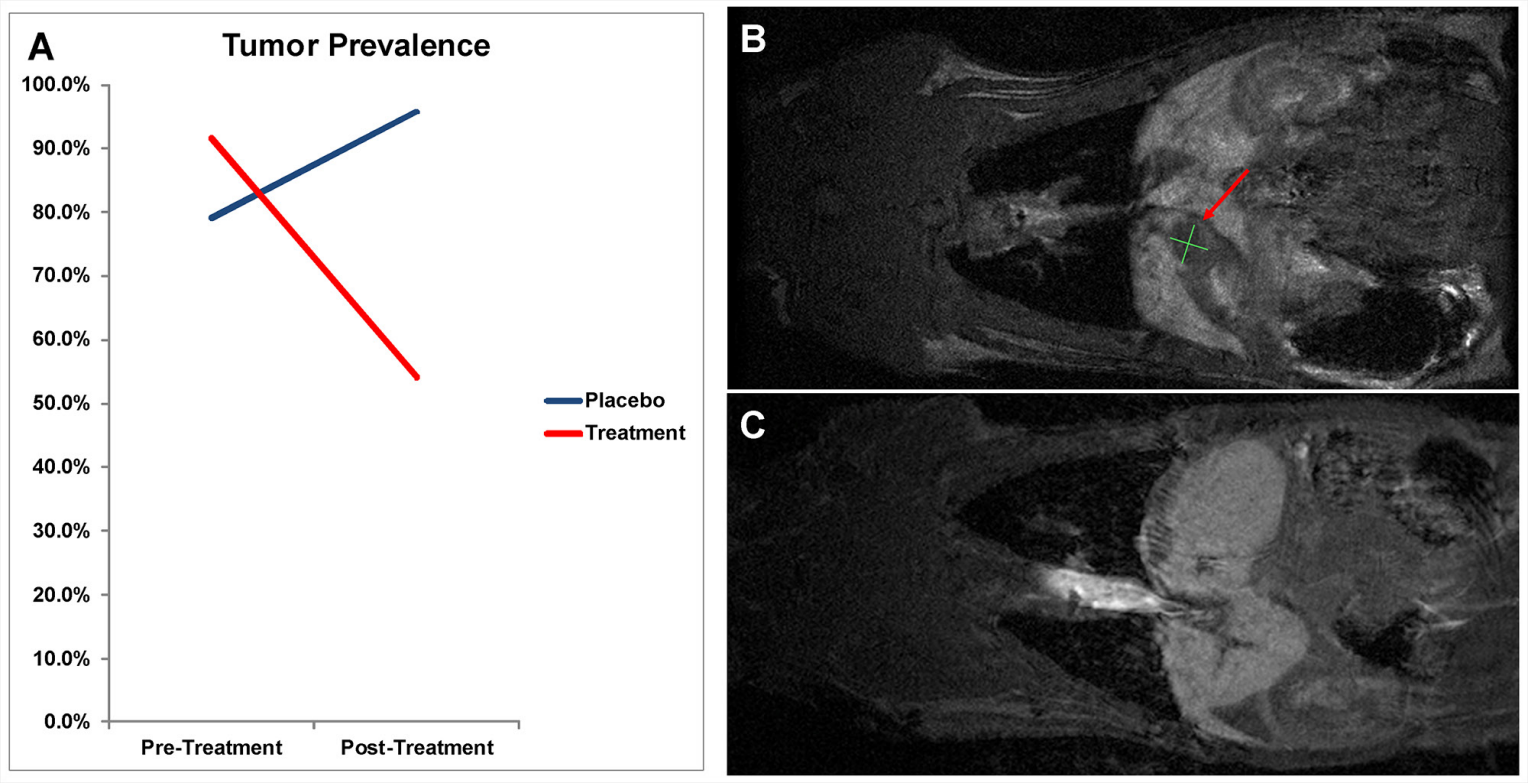
**(A)** Tumor prevalence change by MRI analysis. On comparison of pre and post-treatment MRI between Placebo and Treatment groups, animals receiving abemaciclib demonstrated a decrease in tumor prevalence by 37.5%; whereas control animals displayed an increase in prevalence by 16.7% (p<0.01). **(B,C)** MRI Tumor Response. Pre (B) and post (C) treatment MRI scans of a single animal demonstrating complete response on imaging after treatment with abemaciclib. Red arrow indicates tumor area.

**Figure 5:**
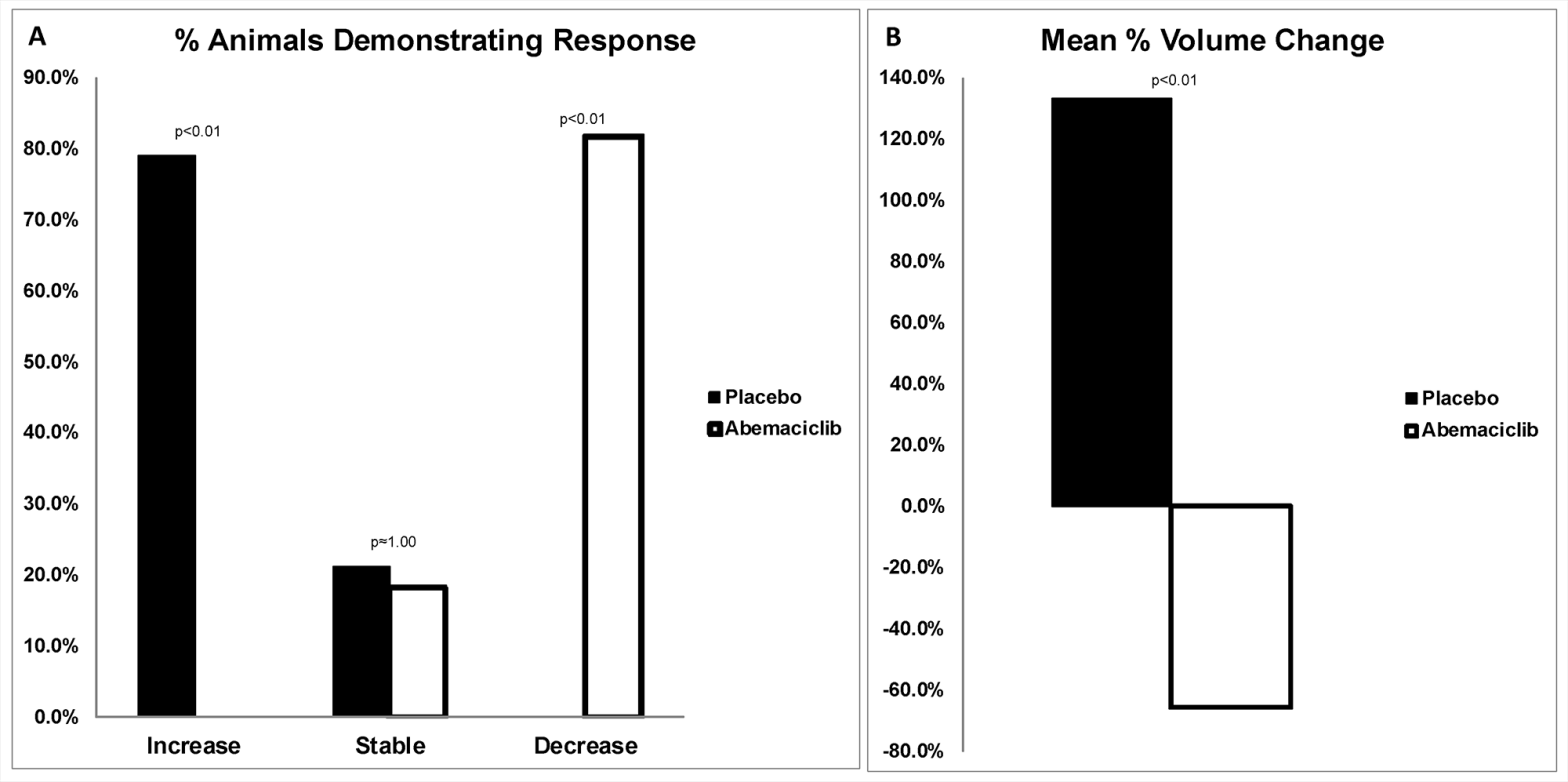
**(A)** Percentage of animals with change in tumor volume. On comparison of pre and post-treatment MRIs, 78.9% of placebo animals demonstrated an increase in tumor volume, and 21.1% had stable disease. Following treatment with abemaciclib, 81.8% of animals demonstrated a decrease in tumor volume, and 18.2% had stable disease. RECIST criteria of <20% increase or <30% decrease in volume were classified as stable. **(B)** Mean percent change in tumor volume. Following treatment, animals in the placebo arm demonstrated a mean increase in tumor volume of 133.5%. Conversely, animals treated with abemaciclib revealed a mean decrease in tumor volume by 65.5% (p<0.01).

Overall, on comparison of pre and post-treatment MRI scans, prevalence increased in the placebo group from 79.2% to 95.8%; whereas prevalence decreased with abemaciclib treatment from 91.7% to 54.2% (p<0.01) (Figure 4). In the placebo group, 78.9% of rats revealed an increase in tumor volume, and the remaining 21.1% had stable disease. Following treatment with abemaciclib, 81.8% of rats demonstrated a decrease in tumor volume, and the remaining 18.2% had stable disease (Figure 5A). The mean percent change in tumor volume increased by 133.5% in the placebo arm and decreased by 65.5% in the treatment arm (p<0.01) (Figure 5B). Endpoint histopathological analysis confirmed an increase of 33.7% well-differentiated EAC tumors in placebo versus treatment animals (p=0.023).

**Figure 6:**
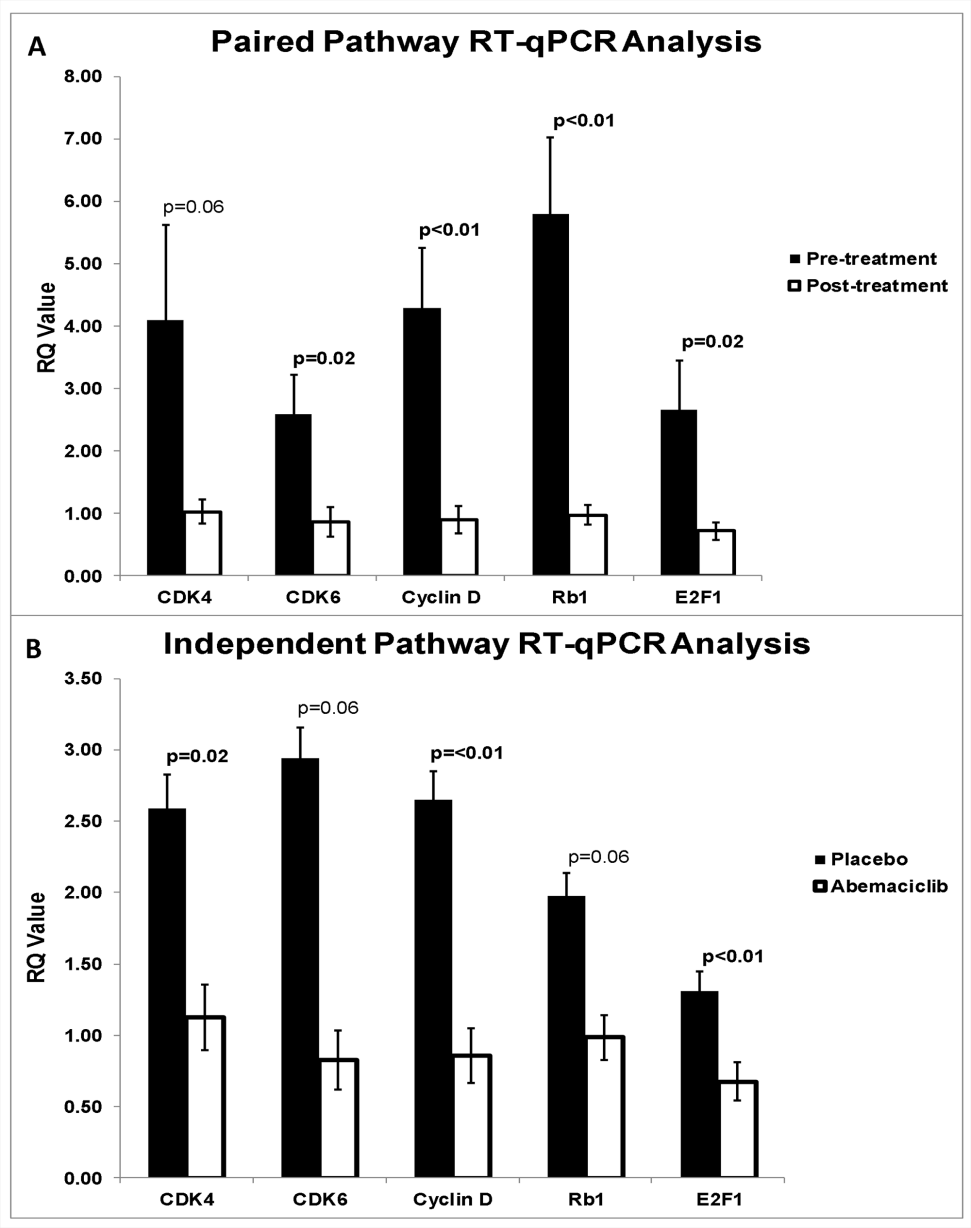
**(A)** qRT-PCR pre versus post treatment pathway analysis. Pre-treatment biopsy samples were compared with harvested samples post-abemaciclib administration. Paired analysis demonstrated downregulation of CDK4 (p=0.057), CDK6 (p=0.017), Cyclin D (p<0.01), Rb1 (p<0.01), and E2F1 (p=0.025). **(B)** qRT-PCR placebo versus abemaciclib pathway analysis. Endpoint placebo and abemaciclib treated samples were compared. Independent analysis demonstrated downregulation of CDK4 (p=0.016), CDK6 (p=0.064), Cyclin D (p<0.01), Rb1 (p=0.56), and E2F1 (p<0.01).

Analysis of CDK4/6 pathway gene expression in pre (n=23) and post-abemaciclib (n=23) samples demonstrated downregulation of CDK4 (p=0.057), CDK6 (p=0.017), Cyclin D (p<0.01), Rb1 (p<0.01), and E2F1 (p=0.025). (Figure 6A). Comparison of post-treatment samples between placebo (n=25) and abemaciclib (n=26) cohorts demonstrated downregulation of CDK4 (p=0.016), CDK6 (p=0.064), Cyclin D (p<0.01), Rb1 (p=0.056), and E2F1 (p<0.01) (Figure 6B). Additionally, endpoint PD-L1 gene expression in the abemaciclib arm was significantly downregulated when compared to pre-treatment samples (p<0.01) and to the placebo arm (p=0.018).

## DISCUSSION

For the first time, the present study demonstrates the efficacy of the CDK4/6 dual inhibitor, abemaciclib, for the treatment of EAC. Abemaciclib displays *in vitro* antitumor activity through increased apoptosis and reduced proliferation with associated pathway inhibition of Cyclin D1, E2F1, p-pRb, and Cyclin A2. Additionally, *in vivo* abemaciclib leads to a substantial reduction in tumor volume through downregulation of CDK4, CDK6, Cyclin D, Rb1, and E2F1 gene expression.

Previous studies have verified that the CDK4/6 pathway inhibition profile is marked by the downregulation of CDK4, CDK6, Cyclin D1, Rb1, p-pRb, E2F1, and Cyclin A2 [19, 20]. In the current study, both *in vitro* Western blot analysis and *in vivo* gene expression levels were validated by the anticipated regulatory effects. *In vitro*, flow cytometry analysis demonstrated an increase in total apoptosis and early apoptosis, revealing true induction of tumor cells. BrdU ELISA demonstrated significant reduction of proliferation in OE33 and FLO1 cell lines, and Western blot confirmed pathway inhibition through reduction of all markers across all cell lines.

*In vivo*, MRI was utilized to compare pre and post treatment tumor volume, with each animal serving as its own control. Prevalence increased in the placebo cohort by 16.7%; however, treatment with abemaciclib decreased prevalence by 37.5%. In other words, 9 animals out of 24 animals demonstrated a complete response, revealing undetectable tumor after 4 weeks of treatment. Moreover, no animals in the treatment cohort demonstrated progressive disease, revealing the possible protective effect of abemaciclib in the prevention of EAC development. Specifically, all tumors in the placebo group either remained stable or increased in volume by a mean factor of 133.5%; whereas, all tumors treated with abemaciclib either reduced in volume or remained stable with a mean volume decrease of 65.5%.

The imaging results were further validated through gene expression evaluation of CDK4, CDK6, Cyclin D, Rb1, and E2F1. Evaluation of post-treatment samples demonstrated significant downregulation of all pathway markers, when compared to pre-treatment and/or placebo. Phosphorylation of Rb1 by the CDK4/6-Cyclin D complex and subsequent activation of E2F1 commits the cell past the G1 checkpoint into S-phase. Therefore, decreased expression of these downstream markers following treatment reveals the mechanistic specificity of abemaciclib, as previously confirmed by preclinical studies of colorectal cancer and melanoma [21, 22].

Limitations of the current study included drug delivery methodology. Clinically, abemaciclib is administered orally; however, oral delivery is incompatible with the modified Levrat model due to bypassing of the stomach and continuous GDER. Therefore, formulation and dosage was adjusted for IP delivery. IP catheterization was performed to mitigate administration-associated complications and clearly determine drug-associated observations. Six animals restricted to the abemaciclib cohort experienced mortality prior to endpoint. The most common complications observed across both the placebo and treatment arms were peritonitis and diarrhea. It was not possible to elucidate if the diarrhea was a direct toxicity effect or a secondary effect of the peritonitis; however, it may be of importance to note that clinical studies of abemaciclib to date have reported diarrhea, neutropenia, nausea, and fatigue as common adverse events [14, 15]. It is likely that the observed peritonitis in this study was related to the direct administration of the drug into the abdominal cavity, due to the lack of similarly reported clinical effects.

The present study demonstrates compelling comprehensive antitumor activity of abemaciclib both *in vitro* and *in vivo* to set the stage for further investigation of the targeted agent in EAC clinical trials. Current treatment options are very limited, signifying an urgent need for the movement of promising novel agents in early and late-line clinical settings [23]. To date, abemaciclib is under investigation for the treatment of multiple cancer types, including breast, pancreatic, and non-small cell lung cancer (NSCLC) [24]. Although additional selective CDK4/6 inhibitors are available, such as palbociclib and ribociclib, all three major compounds display comparable activity and efficacy results in both preclinical and clinical settings [25].

Early preclinical and clinical evidence suggests a potential treatment strategy may be to combine abemaciclib with immunotherapy. Recently, Dempsey, et al demonstrated that abemaciclib and anti-PD-L1 antibody combination resulted in complete tumor regression in 50-60% of immunocompetent mice, compared to 0% with PD-L1 monotherapy, and the induction of immunological memory [26]. Additionally, anti PD-1/PD-L1 pathway agents have demonstrated early promising data for gastroesophageal cancers in Keynote-012 (NCT01848834) and Checkmate 032 (NCT01928394) trials [27, 28]. Interestingly, we recently demonstrated the marked upregulation of the immune microenvironment, including PD-L1, following radiation in both human and rat esophageal cancer [29]. Through the current study, we provided further evidence to suggest cross-regulation between the CDK4/6 and PD-L1 pathways through the significant upregulation of PD-L1 gene expression following abemaciclib treatment. The respective synergistic pathways may represent an ideal combinatorial anti-tumor strategy well-suited for further preclinical or clinical testing in gastroesophageal cancers. Alternatively, another logical potential strategy for clinical deployment may be to test abamaciclib with other approved and syntergistic targeted therapies, such as the VEGF inhibitor Cyramza^®^ in advanced metastatic settings.

The presented study provides a platform for the expedited translation of abemaciclib into clinical trials for the treatment of early stage EAC, based on substantial preclinical activity both *in vitro* and *in vivo* with validated downstream molecular correlate activity. We previously performed comparable studies to evaluate therapeutics for the treatment of EAC, such as hedgehog, heat shock protein 90 (Hsp90), and phosphatidylinositol 3-kinase/mammalian target of rapamycin (PI3K/mTOR) pathway inhibitors; however, CDK4/6 inhibition clearly provides maximal efficacy with minimal toxicity [30–33]. Overall, the rising incidence of EAC and extremely high mortality rates present an emergent need for the development of novel therapeutics to provide durable and meaningful clinical responses. Based on the results of this study, abemaciclib serves as an ideal candidate to provide a novel treatment option in a space where there has been very limited progress to date.

## MATERIALS AND METHODS

### In vitro

#### Experimental design

Efficacy and associated pathway regulation of abemaciclib was evaluated in EAC cell lines OE19, OE33, and FLO1. Median effective dose concentration (ED_50_) was determined using ELISA of WST-1, and apoptosis and proliferation assays were performed using flow cytometry and ELISA of BrdU, respectively. Pathway regulation of p16, CDK4, CDK6, Cyclin D, Rb1, pRB1, E2F1, and Cyclin A2 were evaluated through Western blot.

#### Cell lines

OE19 (JROECL19) and FLO1 (FLO) EAC cell lines were purchased from Sigma-Aldrich (St. Louis, MO) and authenticated in September 2015 through Genetica Cell Line Testing (Burlington, NC). OE33 (JROECL33) was purchased from Sigma-Aldrich (St. Louis, MO) in September 2015 with a validated certificate of authenticity. OE19 and OE33 cell lines were maintained, as previously described by Zaidi et al [31]. FLO1 cells were maintained in Dulbecco’s Modified Eagle’s medium (DMEM) with L-glutamine (Life Technologies, Grand Island, NY; 11965092) and supplemented with 10% Fetal Bovine Serum (FBS) (Life Technologies, Grand Island, NY; 26140079).

#### Median effective dose concentration

Abemaciclib was provided to the Allegheny Health Network under a material transfer agreement (MTA) by ELI Lilly and Company (Indianapolis, IN) in a powder form and prepared in sterile dimethyl sulfoxide (DMSO) at 10mM stock concentration. To determine ED_50_, ELISA-based WST-1 Assay Kit (Millipore Corporation, Billerica, MA; 2210) was utilized. OE19, OE33, and FLO1 cells were exposed to 0-100μM of abemaciclib for 24h. Following exposure, cells were incubated with WST-1 solution at 37°C with 5% CO_2_ humidified air. Absorbance was measured between 30-120min at 440nm with a reference wavelength of 630nm. All experiments were performed in technical triplicates.

#### Detection of apoptosis using flow cytometry

Cell lines OE19, OE33, and FLO1 were utilized to evaluate the effects of abemaciclib on apoptosis through flow cytometry analysis using Annexin-V and Calcein to stratify total and early apoptotic fractions, respectively. Cells were seeded in 6-well plates and treated with the respective ED_50_ dose or left untreated for 24h. Cells were harvested by trypsinization, and the cell pellet was collected. Next, cells were re-suspended in media at a density of 1x10^3^ cells/μL. The cell suspension was centrifuged at 125 x g for 7min at 4°C to remove media and resuspended in 1X binding buffer (Abcam, Cambridge, MA; ab14190). 5μL of Annexin-V-Biotin (Abcam, Cambridge, MA; ab14190) was added to 100μL of cell solution and incubated at room temperature for 10min. Samples were re-centrifuged at 1200 x g for 5min at room temperature, and supernatant was removed followed by re-suspension in 100μL of 1X binding buffer containing 1μg/mL of streptavidin-Cy5 conjugate (Life Technologies, Carlsbad, CA; SA1011) and 1μM calcein-AM (Abcam, Cambridge, MA; ab141420) and incubated at room temperature for 10min. Supernatant was removed as described above. Finally, cells were re-suspended in 50μL of cell buffer solution and mixed by pipetting. The cell chip was prepared according to Agilent Cell Assay Kit protocol (Agilent, Santa Clara, CA; 5067-1519). The chip was immediately evaluated using the Agilent 2100 Bioanalyzer program “Apoptosis Series II” (Agilent, Santa Clara, CA). Treated cells were evaluated in technical triplicates.

#### Cell proliferation analysis

Quantitative analysis of cell proliferation for OE19, OE33, and FLO1 was performed using ELISA BrdU Assay Kit (Roche Applied-Science, Branford, CT; 11669915001). All cells were seeded in a 96-well plate at a density of 2000 cells per well. Cells were treated with abemaciclib at the respective ED_50_ or left untreated and incubated at 37°C with 5% CO_2_ humidified air for 24h. ELISA of BrdU was performed according to manufacturer’s instructions, as previously described [31]. BrdU substrate solution was applied to the cells for 5-30min, and absorbance was measured at 370nm with a reference wavelength of 490nm. All experiments were performed in technical triplicates.

#### Protein analysis

Western blot analysis was performed to semi-quantitatively analyze CDK4/6 pathway expression. Cells were treated with the respective ED_50_ dose or left untreated for 24h. Protein isolation, protein quantification, and Western blot protocol was performed, according to established methodologies [32]. 40μg of protein was utilized, and primary antibodies included Cyclin A2 (Abcam, Cambridge, MA; ab137769), E2F1 (Cell Signaling Technologies, Boston, MA; 3742), Cyclin D1 (Cell Signaling Technologies, Boston, MA; 2978), and phospho-Rb (Ser780) (Cell Signaling Technologies, Boston, MA; 9307). All membranes were incubated with the respective primary antibodies at a 1:1000 dilution. GAPDH antibody (Cell Signaling Technologies, Boston, MA; 5174) was used as a loading control at 1:1000. Finally, signals were developed using a chemiluminescence reagent (BIO-RAD, Hercules, CA; 170-5060).

### In vivo

#### Experimental design

Modified Levrat surgery of end-to-side esophagojejunostomy was performed on 57 rats to induce GDER and progression to EAC as described by Gibson et al [30]. At 36 weeks post-surgery, all animals received an MRI and endoscopic biopsy to determine initial tumor volume and baseline pathway expression, as previously described [32]. Intraperitoneal (IP) catheterization and port placement was performed (Supplementary Figure 1), and all animals were randomized into placebo or treatment arms (Figure 1B). Placebo or abemaciclib was administered daily at 26mg/kg for 28 days through IP catheters. Within 24 hours of the last treatment dose, all animals received a final MRI to determine endpoint tumor volume and were euthanized for esophageal harvest. Efficacy of abemaciclib was determined through the comparison of pre and post-treatment tumor volumes of each animal, change in MRI prevalence between control and treatment groups, quantitative real-time polymerase chain reaction (qRT-PCR) analysis of CDK4/6 pathway expression between pre-treatment biopsy specimens and endpoint harvested samples, and evaluation of gross histopathology. MRI imaging response was scored according to the clinical gold standard of response evaluation criteria in solid tumors (RECIST version 1.1); Progressive disease: ≥20% increase in volume or detection of a new mass; Partial response: ≥30% decrease in volume; Complete response: No remaining evidence of disease; Stable disease: <20% increase or <30% decrease [34]. Lastly, PD-L1 gene expression was evaluated to determine potential cross-regulation between cell cycle checkpoint and immunological markers.

#### Drug formulation and administration

Use and preparation of abemaciclib was performed under the guidance of the Institutional Biosafety Committee (IBC) of Allegheny General Hospital in Pittsburgh, Pennsylvania under protocol #110. Abemaciclib was prepared weekly under sterile conditions as per the manufacturer’s guidelines in a suspension of 50mM acetate buffer pH 4 (Ricca Chemical Company, Arlington, TX; #R0048050-4A) at a concentration of 8 mg/mL and stored at 4°C. Abemaciclib solution or placebo (acetate buffer) was administered through IP port injection daily.

#### Gene expression analysis

RT-qPCR was performed to evaluate CDK4/6 pathway and PD-L1 gene expression as previously described [35]. In brief, esophageal tissues were macrodissected, RNA was isolated and reverse transcribed, and RT-qPCR was performed. Real-time PCR reactions were conducted at 95°C for 15 minutes, followed by 40 cycles of 94°C for 15 seconds, 55°C for 30 seconds, and 70°C for 30 seconds, using a StepOnePlus real-time quantitative system (Applied Biosystems; Carlsbad, CA). Raw data was exported from the real-time instrument software and relative gene expression was calculated using the DD-Ct method. Specific antibodies included CDK4 (Qiagen, Valencia, CA, # PPR06455B-200), CDK6 (Qiagen, Valencia, CA, # PPR50657A), Cyclin D (Qiagen, Valencia, CA, # PPR06517C), Rb1 (Qiagen, Valencia, CA, # PPR06558A-200), E2F1 (Qiagen, Valencia, CA, # PPR55684A-200), and PD-L1 (Cd274) (Qiagen, Valencia, CA, #PPR65311A). Endogenous controls were B-Actin (Qiagen, Valencia, CA, #PPR06570C) and RPLP1 (Qiagen, Valencia, CA, #PPR42363C).

#### Statistical analysis

Statistical analysis was performed using SPSS software (IBM, Armonk, NY; Version 21). An independent two-tailed *t* test was utilized for comparison of mean percent change in tumor volume between placebo and treatment groups. A paired two-tailed *t* test was performed to evaluate pre versus post treatment gene expression. Fisher’s exact tests were used for comparison of animal response (increased, stable, or decreased) and change in endpoint tumor prevalence between placebo and treatment groups. A p<0.05 was considered to be statistically significant.

## SUPPLEMENTARY MATERIALS FIGURE


