# Supplementary material for: CDK4/6 dual inhibitor abemaciclib demonstrates compelling preclinical activity against esophageal adenocarcinoma: a novel therapeutic option for a deadly disease
Source: Oncotarget. 2017 Nov 1;8(59):100421–32. doi: 10.18632/oncotarget.22244 (PMC5725031; doi:10.18632/oncotarget.22244)
Supplement: Supplementary file 1 [file oncotarget-08-100421-s001.pdf]

CDK4/6 dual inhibitor abemaciclib demonstrates compelling preclinical activity against esophageal adenocarcinoma: a novel therapeutic option for a deadly disease

SUPPLEMENTARY MATERIALS

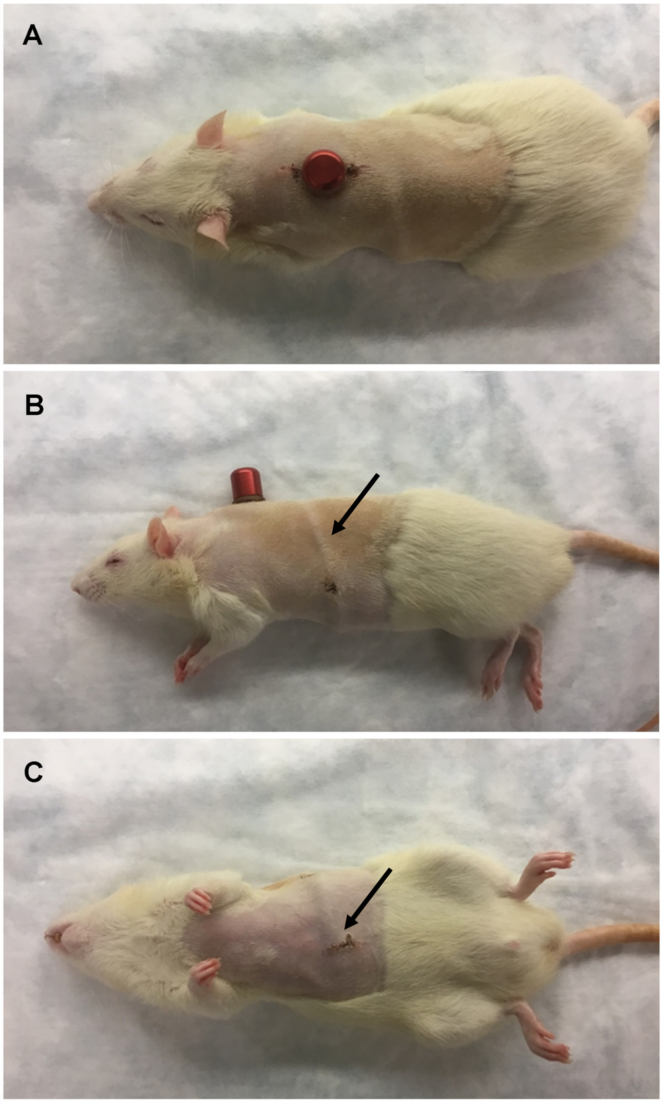

Supplemental Figure 1: Intraperitoneal catheterization and port placement. 3Fr 25cm round-tip catheters (Instech Labs, Plymouth Meeting, PA; C30PU-RFV1308) with 22ga external ports (Insteach Labs, Plymouth Meeting, PA; VAB95BS) were utilized as intraperitoneal catheters for administration of placebo and treatment agents with specialized injectors (Instech Labs, Plymouth Meeting, PA; VAH6M). All animals were anesthetized with isoflurane, and sterility was maintained throughout the procedure. Briefly, rats were placed supine, and a small incision was made through the abdominal wall. The round-tip catheter was directed into the lower right abdominal quadrant and secured to the abdominal wall with 4-0 vicryl. The loose catheter was tunneled subcutaneously and externalized through an intermediate incision on the left flank, the abdominal incision was closed, and the animal was rotated to a prone position. A larger incision was made in the dorsal-scapular region, and the catheter was tunneled subcutaneously, externalized, and secured to the port. The intermediate incision and skin around the port was closed with 4-0 vicryl, and antibiotic ointment was placed on all incisions to prevent infection. Protective aluminum caps (Instech Labs, Plymouth Meeting, PA; VAB95CAP) were maintained over the port to protect sterility and allow for pair housing of the rats. All animals received 3 days of ketoprofen and enrofloxacin for pain management and infection prevention.
